# 1330. Clinical Associations and Trajectory of “Long COVID”

**DOI:** 10.1093/ofid/ofab466.1522

**Published:** 2021-12-04

**Authors:** Karen Jacobson, Vidhya Balasubramanian, Hector F Bonilla, Martina Madrigal, Isabelle Hack, Natasha Purington, Upinder Singh, Haley Hedlin, Prasanna Jagannathan

**Affiliations:** 1 Stanford University, Stanford, California; 2 Stanford University School of Medicine, Palo Alto, California

## Abstract

**Background:**

Persistent symptoms after acute COVID-19 are being increasingly reported. To date, little is known about the cause, clinical associations, and trajectory of “Long COVID”.

**Methods:**

Participants of an outpatient clinical trial of Peginterferon-Lambda as treatment for uncomplicated SARS-CoV-2 infection were invited to long term follow-up visits 4, 7, and 10 months after initial COVID-19 diagnosis. Ongoing symptoms and functional impairment measures (work productivity and activity index (WPAI), NIH toolbox smell test, 6-minute walk test) were assessed and blood samples obtained. “Long COVID” was defined as presence of 2 or more typical symptoms (fatigue, hyposmia/hypogeusia, dyspnea, cough, palpitations, memory problems, joint pain) at follow up. Associations between baseline characteristics, initial COVID-19 clinical course, and presence of “Long COVID” during follow-up were assessed using generalized estimating equations accounting for repeated measurements within individuals.

**Results:**

Eighty-seven participants returned for at least one follow-up visit. At four months, 29 (34.1%) had “Long COVID”; 19 (24.7%) met criteria at 7 months and 18 (23.4%) at 10 months (Figure 1). Presence of “Long COVID” symptoms did not correlate significantly with functional impairment measures. Female gender (OR 3.01, 95% CI 1.37-6.61) and having gastrointestinal symptoms during acute COVID-19 illness (OR 5.37, 95% CI 1.02-28.18) were associated with “Long COVID” during follow-up (Figure 2). No significant associations with baseline immunologic signatures were observed.

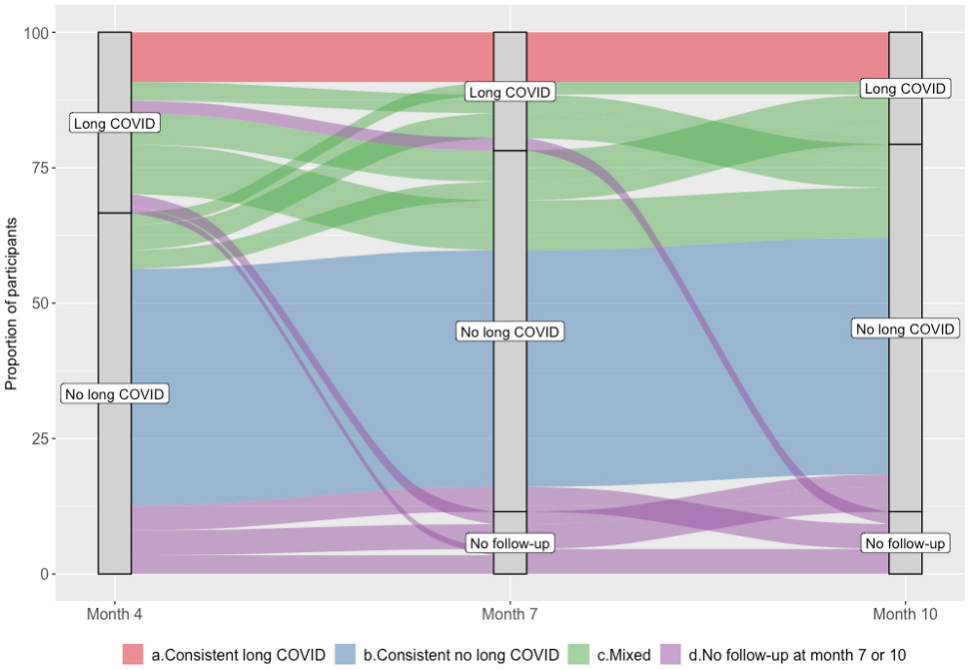

Figure 1. Alluvial plot of long term follow-up participants showing outcomes of symptoms at each visit.

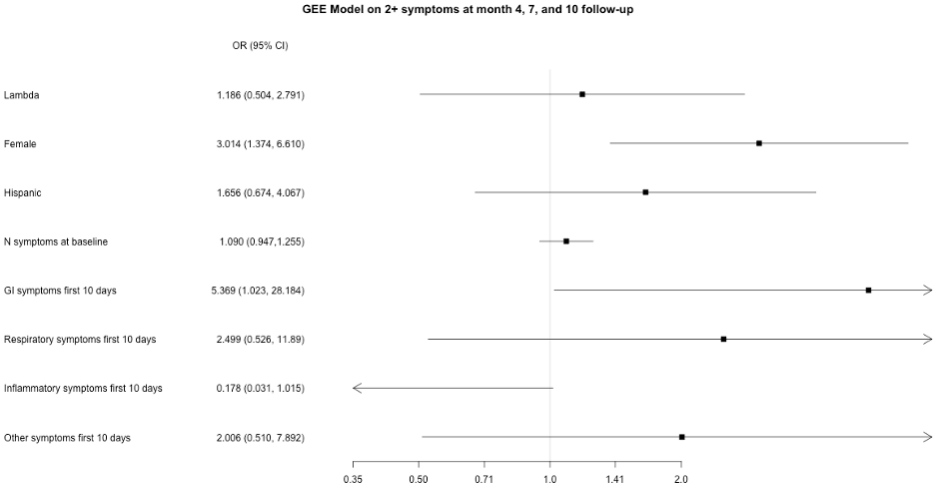

Figure 2. Generalized Estimating Equations Model showing associations with “Long COVID” (presence of 2+ symptoms) at month 4, 7, and 10 following acute infection using unstructured correlation matrix.

**Conclusion:**

“Long COVID” was prevalent in this outpatient trial cohort and had low rates of resolution over 10 months of follow up. Female sex and gastrointestinal symptoms during acute illness were associated with “Long COVID”. Identifying modifiable risk factors associated with the development of persistent symptoms following SARS-CoV-2 infection remains a critical need.

**Disclosures:**

**All Authors**: No reported disclosures

